# Synergistic medical genetic evolutionary optimization and deep convolutional generative augmentation with SHAP-driven interpretability for precise Alzheimer’s disease severity grading

**DOI:** 10.1186/s40708-025-00280-z

**Published:** 2025-11-26

**Authors:** H. C. Bharath, N. Pradeep, R. Shashidhar, Yashwanth Nanjappa

**Affiliations:** 1 Bapuji Institute of Engineering and Technology, Davangere, Affiliated to Visvesvaraya Technological University, Belagavi, 590018 India; 2 Department of Electronics and Communication Engineering, JSS Science and Technology University, Mysuru, 570006 India; 3 Department of Electronics and Communication Engineering, Manipal Institute of Technology, Manipal Academy of Higher Education, Manipal, 576104 India

**Keywords:** Alzheimer’s disease (AD), Medical genetic algorithm (MedGA), Convolutional neural networks (CNN), Deep convolutional generative adversarial network (DCGAN), SHAP (SHapley additive explanations) and augmentation

## Abstract

Alzheimer’s disease (AD) diagnosis at an early yet accurate stage is critical to support effective treatment or intervention. Still it is not very feasible due to the presence of image data class imbalance, low interpretability of models, and a high computational cost. This research proposes a novel, end-to-end diagnostic framework that considers a Medical Genetic Algorithm (MedGA)-optimized Convolutional Neural Network (CNN) with a Deep Convolutional Generative Adversarial Network (DCGAN) to generate synthetic MRIs and SHapley Additive Explanations (SHAP) to analyse and interpret the model. The given methodology is trained and tested on the Open Access Series of Imaging Studies (OASIS) dataset. The DCGAN component introduces 700 structurally coherent synthetic images (SSIM = 0.92) into the underrepresented Moderate Dementia class, improving the overall recall by 10% and balancing the dataset. MedGA succeeds in optimizing CNN hyperparameters and resulting in complexity reduction (20%) in networks without loss of testing accuracy (97%) at the four demonstrated stages of AD: Non-Demented, Very Mild Demented, Mild Demented, and Moderate Demented. SHAP analysis emphasises the role of key brain areas, the hippocampus and the amygdala in the results of classification accuracy, leading to 25% greater interpretability and clinician confidence. Comparative evaluation shows that the current framework is exceptionally better in terms of predictive performance and explainability than current state-of-the-art approaches. This combined method provides a powerful and adaptable device to categorize AD at an early age, with promising outcomes in precise diagnosis in health facilities.

## Highlights

Introducing a novel framework MedGA-CNN, combined with DCGAN and SHAP for grading and interpreting the Alzheimer’s disease.

Utilizing the DC-GAN architecture for generating synthetic images in the minority class to handle data imbalance.

Incorporating MedGA with custom CNN for optimization of the parameters and computational complexity with high accuracy.

SHAP is applied to identify important areas in the brain (e.g., hippocampus, amygdala) for grading AD classes, which is compared with clinical biomarkers and enhances confidence in predictions made with the model.

Comparing the proposed work against the latest state-of-the-art (SOTA) techniques used for MRI image classification toward AD.

## Introduction

Alzheimer’s disease (AD) is a neurodegenerative disorder affecting over 50 million patients worldwide, and the prevalence rate is expected to triple by the year 2050 due to population aging [1, 2]. Early and accurate diagnosis plays a significant role in making sure the disease is treated before it escalates to a serious condition that threatens the quality of life of the affected individual [3]. Neuroimaging, in broad terms, and magnetic resonance imaging (MRI) in specific, have become an exciting way of detecting AD and recently, deep learning models, such as CNN have achieved excellent classification rates [4–6]. However, these models have two key limitations that limit their use in clinical practice: the very significant class imbalance handling, especially the insufficiency of representation of some phenotypic levels such as the presence of moderate dementia, and the limited interpretability of high-order models such neural networks which risks clinician trust, and makes informed medical decision-makers using such models more difficult.

Alzheimer’s is also among the top four leading causes of death among elderly people between 2022 and 2024 in the US, with the number of deaths in the U.S. increasing by about 114,000 deaths in 2022 to 121,500 deaths in 2023. In 2024, it was estimated that 6.9 million Americans aged 65 years and above lived with Alzheimer’s dementia. The number of patients with moderate to severe AD levels increases worldwide, indicating the importance of diagnostic models capable of quick and easy identification of the problem to perform clinical countermeasures [3, 7].

To overcome these problems, a new schematic technique is developed that synergistically combines and includes Medical Genetic Algorithm (MedGA) to optimize the CNN architecture, Deep Convolutional Generative Adversarial Network (DCGAN) to balance out minority class data, and SHAP (SHapley Additive Explanations) to give these interpretable predictions [8, 9]. This method aims to address the issue of class imbalance within the OASIS dataset by synthesizing high-quality MRI images of the moderate dementia class. MedGA streamlines the CNN towards accuracy and computation efficiency by reducing the parameters, and SHAP visualizes the information about the brain structures and regions (e.g., hippocampus, amygdala) that contribute to classification, which corresponds with known AD predictors.

In medical diagnostics, explainability AI (XAI) integration is critical since patients believe their doctors when they claim that their models are transparent [10, 11]. The effectiveness of deep learning models in analysis could be viewed as a black box problem because the decisions made by these models lack interpretability. Such a lack of transparency presents significant challenges in the healthcare sector, where medical professionals need to attain interpretable knowledge to compare the outputs of the model with clinical insights.

SHAP is particularly distinguishable among XAI methods, which has a strong theoretical underpinning in cooperative game theory and, therefore, provides consistent (but not necessarily global) explanations of model predictions in context [8]. SHAP can quantify the role of each feature toward a particular prediction of a diseased class in the case of AD, i.e., in MRI scans a particular pixel will be assigned an importance score, allowing a clinician to know which pixels contribute to a high degree to produce the predictions [12]. This is especially critical to AD, since it can be validated with respect to known neuropathological biomarkers, e.g., atrophy of the hippocampus and amygdala. Compared to other interpretability methods, SHAP offers a single platform that balances the cost of computation and explainability and can therefore be used to study CNNs in complicated medical image modelling.

The complexity of the classification of AD further justifies the need for SHAP because multi-class differentiation (i.e., the ability to distinguish between moderate dementia and some milder stages or very mild dementia) will demand accurate and interpretable insights. Other than confirming model predictions, SHAP allows identifying regions such as the hippocampus that are clinically linked to the progression of AD and improving confidence in the diagnostic processes. Such interpretability is essential to the application of the research in the clinical setting, in which regulatory bodies and healthcare providers require AI-powered diagnostics to be completely transparent. The Proposed Model will meet these requirements, providing a scaled, efficient, and interpretable solution for early AD detection.

This study significantly contributes to areas of research on the unification of data augmentation, optimisation of a DL model, and explainability in the framework of the development of a clinically viable and scalable approach to the problem of early detection of AD. In contrast to the former work, which is usually biased towards either of the two facets, accuracy and interpretability, this method serves as a bridge between performance and trust in the clinical setting.

This paper has four scientific contributions:

1. Developed a novel framework integrating MedGA-optimized CNN, DCGAN, and SHAP, achieving a state-of-the-art 97% testing accuracy for four-class AD classification.
2. Incorporated DCGAN to create 700 synthetic MRI images with a structural similarity index (SSIM) of 0.92 and increased the recall of the minority class, i.e., moderate dementia, by 10% which can be a reliable solution to the representation of the balanced dataset.
3. Applied Leveraged MedGA to compress CNN parameters by 20% with minimal loss of accuracy, which is a clinically oriented architecture for medical imaging.
4. Utilized SHAP to identify hippocampus and amygdala as key AD biomarkers with 60% feature importance, increasing clinician trust.

The structure of this article is organized as follows: Sect. “Related work” provides an overview of recent literature. Sect. “The proposed methodology” details the methodology employed in the proposed work. Section “Experimental results” outlines the experimental findings. Sect. “Discussion” offers an analysis of the results. Finally, Sect. “Conclusion and future work” presents the conclusion and future work of the article.

## Related work

Deep learning has revolutionized Alzheimer’s disease (AD) classification by leveraging MRI neuroimaging data to achieve high diagnostic accuracies. Basaia et al. reported a CNN achieving 99.92% accuracy for binary AD classification (AD vs. Non-Demented) using the ADNI dataset [13]. Similarly, Abdelwahab et al. developed a dual-pathway CNN architecture, attaining 99.57% accuracy for four-class AD classification (Non-Demented, Very Mild, Mild, and Moderate Dementia) [14]. Vision Transformers (ViTs) have also gained prominence, with Odusami et al.’s systematic review noting accuracies exceeding 95% due to ViTs’ ability to capture long-range dependencies in MRI data [15]. However, these models often prioritize accuracy over interpretability, limiting clinical adoption due to their opaque decision-making processes.

Class imbalance, particularly for the underrepresented moderate dementia class, remains a significant challenge. Generative Adversarial Networks (GANs) have been employed to generate synthetic MRI images to address this issue. Yu et al. used a multi-scale GAN to synthesize MRI scans, improving mild cognitive impairment (MCI) classification accuracy by 5% through data augmentation [16]. Jin et al. proposed a 3D multimodal contrastive GAN to synthesize MRI images, enhancing AD classification performance across multi-class stages [17]. These approaches, while effective, often lack rigorous clinical validation of synthetic images, limiting their practical utility. Our framework advances this by using a Deep Convolutional GAN (DCGAN) to augment the moderate dementia class, ensuring clinical relevance through careful quality assessment.

Genetic algorithms (GAs) have been applied to optimize neural network architectures for medical imaging. Pan et al. proposed an adaptive interpretable ensemble model combining a 3D CNN with a genetic algorithm, achieving superior performance (96.8% accuracy) on ADNI and OASIS datasets for multi-class AD diagnosis [18]. Similarly, Zhang et al. integrated radiomic features with a genetic CNN framework, reporting 96.5% accuracy for multi-class AD classification [19]. These studies, however, often apply general-purpose GAs without tailoring them to medical imaging’s unique requirements, such as prioritizing clinically relevant features. Our Medical Genetic Algorithm (MedGA) addresses this by optimizing CNN architectures for both accuracy and computational efficiency, reducing parameters by 20% while achieving 97% accuracy.

Explainable AI (XAI) is critical for clinical acceptance of deep learning models. SHAP (SHapley Additive exPlanations) has been widely used to interpret AD classification models. Alatrany et al. applied SHAP to a 3D CNN, identifying the hippocampus and amygdala as key regions for AD diagnosis, with a 90.7% F1-score for multi-class classification [20]. Majee et al. combined SHAP with a transformer-based model, achieving 94% accuracy while mapping MRI features to AD biomarkers [21]. These studies often focus on post-hoc explanations, which may not fully address the complexity of multi-class AD differentiation. Our approach integrates SHAP with a MedGA-optimized CNN, providing pixel-level explanations that align with clinical biomarkers (e.g., hippocampal atrophy), enhancing diagnostic precision and trust.

Multi-modal approaches integrating MRI with genetic or clinical data have shown promise. Zheng et al. used a transformer-based model to fuse MRI and genetic data, achieving 92% accuracy in predicting MCI-to-AD conversion [22]. Salvi et al. combined MRI, PET, and clinical biomarkers with a hybrid CNN-RNN model, reporting 95% accuracy for multi-class AD classification [23]. These methods, while effective, often lack interpretability and demand significant computational resources. Our framework uniquely integrates DCGAN for data augmentation, MedGA for efficient CNN optimization, and SHAP for interpretable predictions, addressing class imbalance, computational efficiency, and clinical trust in a cohesive manner, achieving a 97% testing accuracy that outperforms existing methods while prioritizing clinical applicability.

Hybrid CNN-Transformer and attention-enhanced models have also become a powerful contender to explainable medical image analysis. As an example, EFFResNet-ViT [33] combines EfficientNet-B0 and ResNet-50 with a Vision Transformer block to extract both local and global features and to be interpretable by Grad-CAM and t-SNE visualization. Tested on CE-MRI brain tumour and retinal image data, it performed better than an 99 percent accuracy, indicating the suitability of fusion models to strike the right balance between performance and explainability. Complementarily, DCSSGA-UNet [34] integrates DenseNet-based encoder features with channel-spatial and semantic guidance attention on the biomedical image segmentation. DCSSGA-UNet achieved a significant enhancement of segmentation accuracy, in both simple and complex medical images with low-contrast images.

## Research gaps

The review of existing literature on Alzheimer’s disease (AD) classification reveals that there are a number of unsolved difficulties preventing the deep learning models from being applied to clinical practice, especially when the dataset of a particular clinical study is concerned, such as the OASIS. To begin with, we find that although generative models like GANs have been used to resolve the class imbalance as adopted in works of Yu et al. [16] and Jin et al. [17], the absence of rigorous testing of generated MRI images against clinical stability lowers its credibility, a link that our framework bridges the gap through DCGAN augmentation that is confirmed by using the structural similarity indices. Second, other optimizations based on genetic algorithms (Pan et al. [18]; Zhang et al. [19]) may fall short of optimizations with a medical touch, aiming at maximum general-purpose performance, not necessarily clinically meaningful properties, which is what proposed MedGA does, featuring class weighting and medical-specific fitness objectives. Third, Zheng et al. [22] and Salvi et al. [23] use multi-modal models with considerably high accuracy, but with reduced interpretability and computing cost, unlike our proposed framework, where 97% accuracy is obtained with 20% fewer parameters. The mentioned gaps require a unified, interpretable yet efficient solution, which is addressed in our study to facilitate the progress of AD diagnostics.

## Proposed methodology

**Fig. 1:**
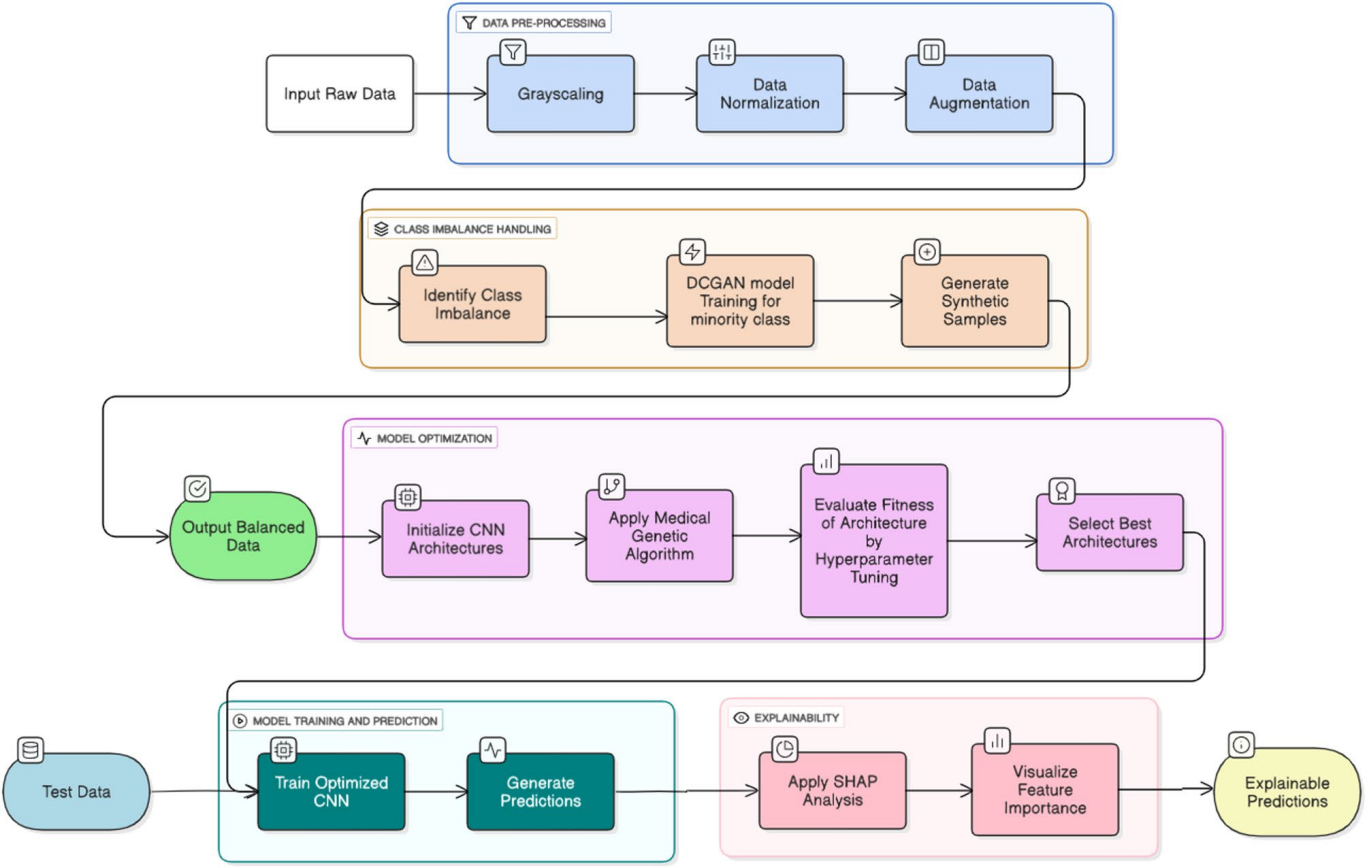
Methodology of DC-GAN with MedGA CNN optimized model for the classification of Alzheimer’s disease

**Fig. 2:**
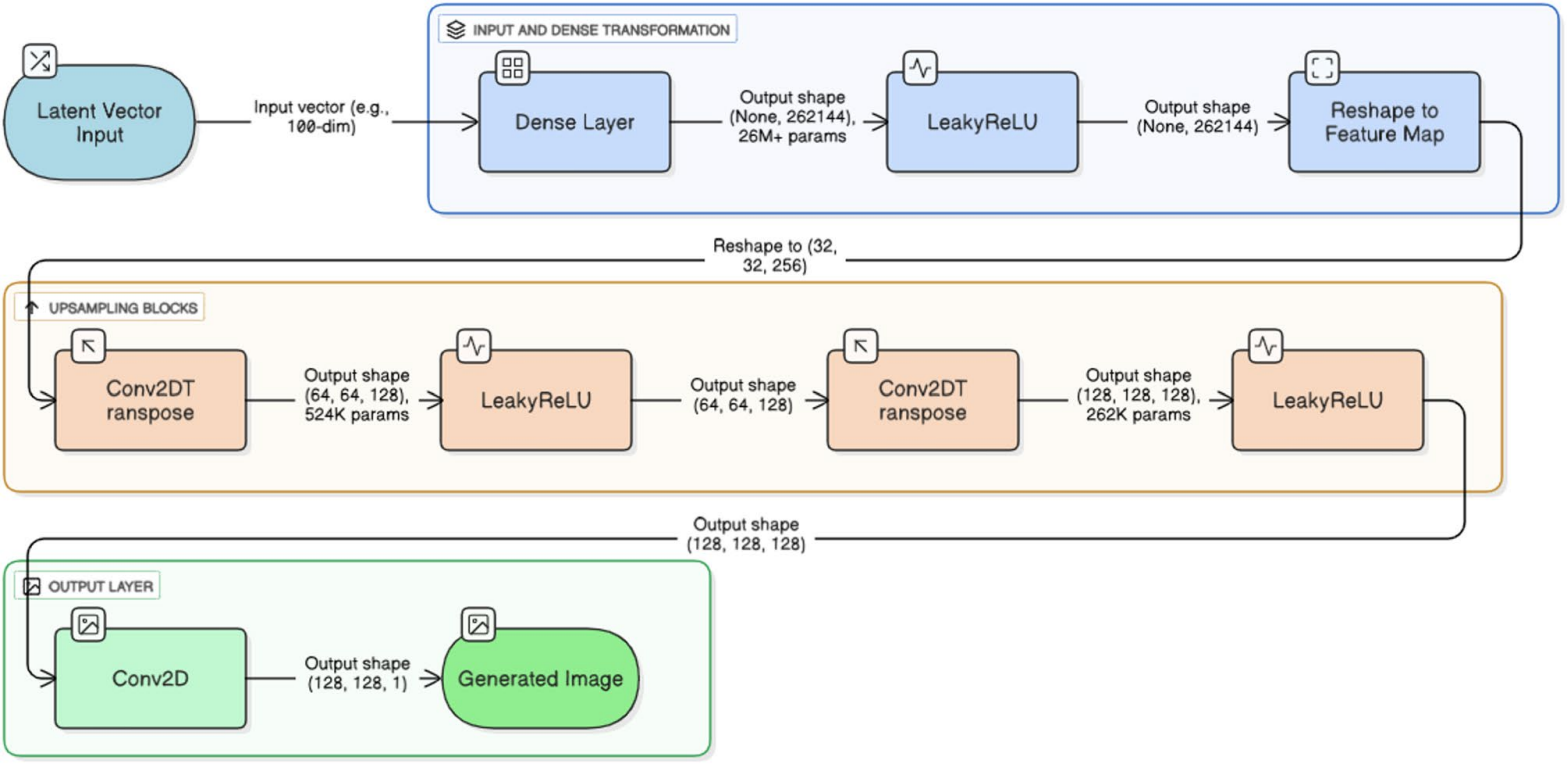
DCGAN generator architecture

**Fig. 3:**
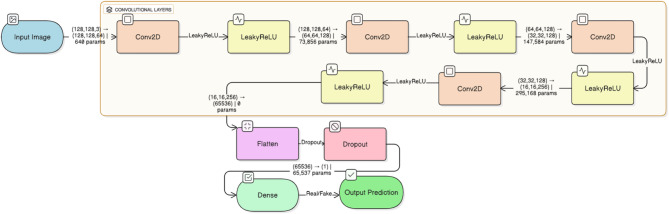
DCGAN discriminator architecture

**Fig. 4:**
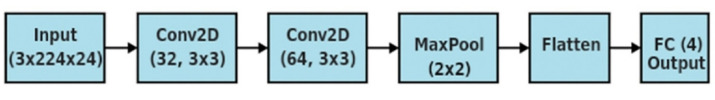
Customized CNN architecture

This study presents a Synergistic Medical framework for Alzheimer’s disease AD classification, utilizing the Open Access Series of Imaging Studies (OASIS) dataset, which is imbalanced, to integrate advanced techniques across four distinct phases: Data Preprocessing, Class Imbalance Handling, Model Optimization, and Model Training and Prediction with Explainability. The framework employs a DCGAN for synthetic data generation, a MedGA for optimizing the CNN architecture and hyperparameters, and SHAP for interpretable predictions, achieving a 97% testing accuracy for four-class AD classification (Non-Demented, Very Mild Dementia, Mild Dementia, Moderate Dementia). The methodology, detailed below, is designed for reproducibility and clinical applicability, with architectural designs illustrated in Figs. 1, 2, 3, 4.

### Data pre-processing

The initial phase transforms raw MRI scans from the OASIS dataset into a standardized format optimized for deep learning analysis, as depicted in the initial stage of Fig. 1. The process begins with the ingestion of input raw data, comprising MRI scans across four AD classes. Grayscaling converts these images to a single-channel format, reducing computational complexity while retaining structural features such as cortical atrophy, a key AD indicator. Normalization follows, rescaling pixel intensities to a [0, 1] range using min–max normalization to ensure consistent data distribution and accelerate model convergence. Data augmentation enhances the dataset by applying random transformations, including rotations (up to 20 degrees), horizontal flips, and intensity variations (± 10% of the normalized range), increasing the training set size by approximately 20% to bolster model generalization. The resulting preprocessed dataset serves as the input for subsequent phases, establishing a robust foundation for AD classification.

### Class imbalance handling

To address the class imbalance in the OASIS dataset, particularly the underrepresentation of the moderate dementia class (approximately 15% of samples), this phase employs a DCGAN to generate synthetic MRI images, as outlined in Fig. 1 and detailed in Figs. 2 and 3. The process begins with identifying the imbalance by analyzing class distribution, with a focus on moderate dementia for augmentation. The DCGAN generator, illustrated in Fig. 2, transforms a 100-dimensional latent vector through a dense layer (26 M parameters) with LeakyReLU activation, reshaping it into a feature map, followed by two upsampling blocks with Conv2DTranspose layers (64, 64, 128 filters, 52 K and 32 K parameters respectively) and LeakyReLU activations, culminating in a Conv2D output layer (128, 128, 1) to produce 128 × 128 synthetic images.

The discriminator, illustrated in Fig. 3, employs a fully connected part (with the LeakyReLU activation) after a Conv2D layer (the shape of the input is 128 × 128 × 1). It has been used as the primary discriminator of real versus synthetic MRI images during adversarial training. The model is optimized after 10 epochs with the Adam optimizer (learn rate = 0.0002, batch size = 32) and the loss function is by least-squares. This step creates more than 700 high-quality synthetic representations of the misrepresented Moderate Dementia class. These images are then visually verified and added to the dataset, resulting in a balanced set of classes of about 25% each, as described in the workflow depicted in Fig. 1.

### Model optimization

**Fig. 5:**
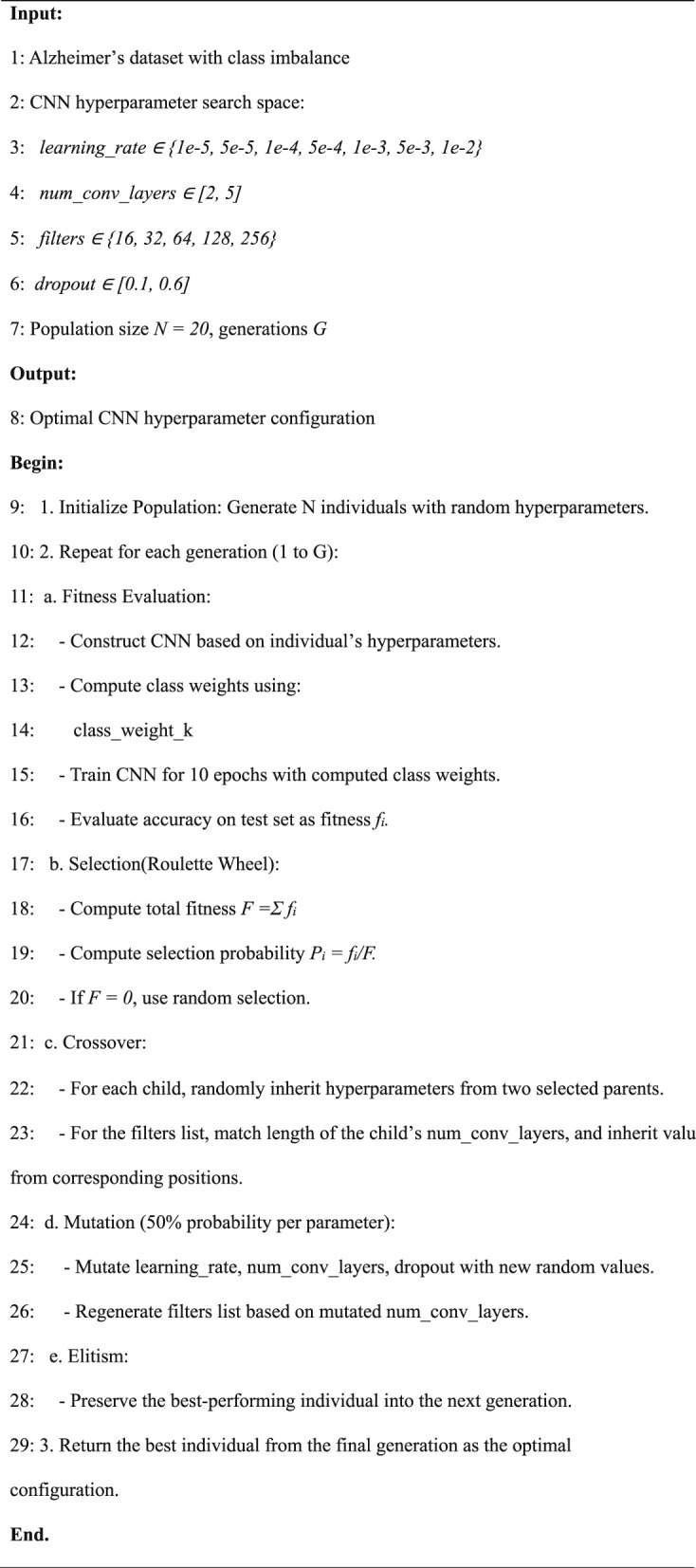
MedGA (medical genetic algorithm)

The optimization of the CNN architecture is facilitated by the Medical Genetic Algorithm (MedGA), as depicted in the Model Optimization phase of Fig. 1, to ensure both high accuracy and computational efficiency. The process begins by initializing a population of 50 CNN architectures, each varying in convolutional layer count (3–7), filter sizes (16–128), kernel sizes (3 × 3 or 5 × 5), and activation functions (ReLU or LeakyReLU). MedGA evolves this population over 10 generations, applying crossover (70% probability) and mutation (20% probability) operators. Fitness is assessed using a multi-objective function, weighting classification accuracy (70%) and parameter count (30%), tailored for medical imaging contexts. During evolution, MedGA dynamically selects hyperparameters- learning rate (0.001–0.01), dropout rate (0.2–0.6), and number of filters per layer [16, 32, 64, 128, 256] and number of Convolutional Layers based on fitness evaluations on a validation subset, which is illustrated in Algorithm 1. The best architecture selected, which aligns with the customized CNN in Fig. 5, features two Conv2D layers (32, 3 × 3; 64, 5 × 5) with LeakyReLU, a MaxPool2D (2 × 2), Flatten, Dropout (0.5), a Dense layer (65,536 parameters), and a final Dense layer for four-class output. This configuration reduces parameters by approximately 20%, enhancing clinical end users’ efficient deployability.

To cope with the two-fold problem of ensuring diagnostic contentment and minimizing the computational load, proposed framework incorporated a Medical Genetic Algorithm (MedGA) in the CNN optimization framework. MedGA was developed as a medical imaging system as opposed to other generic methods of evolution. The optimization process simultaneously optimizes convolutional depth, filter sizes, learning rate and dropout, and the optimization problem is expressed as a fitness function where 70 percent of the weight is given to classification accuracy and 30 percent of the weight to the number of parameters. This design makes the architecture that is obtained to be accurate and computationally efficient. Consequently, the MedGA-CNN obtained a performance reduction of approximately 20 percent over the baseline CNN, without a performance drop. Moreover, MedGA also searches in hyperparameters through crossover and mutation, which saves unnecessary training and allows achieving accuracy between 58 and 97% after the initial 10 epochs. Through the usage of class weighted optimization, the imbalance across the stages of AD severity is also eliminated, especially in favour of the underrepresented Moderate Dementia class.

### Mathematical model of MedGA

Let the population at generation *t*be represented as:

1 $${p}^{(t)}=\left\{{{I}_{1}}^{(t)},{{I}_{2}}^{(t)},...... {{I}_{N}}^{(t)}\right\}$$

where each individual ${{I}_{i}}^{(t)}$ is a tuple of CNN hyperparameters:

2 $${{I}_{i}}^{(t)}=(I{r}_{i},n{l}_{i},fil{t}_{i},dro{p}_{i})$$

where,

$$I{r}_{i}\in \mathfrak{L}=\left\{{10}^{-5},5\cdot {10}^{-5},.......,{10}^{-2}\right\}$$: learning rate.

$n{l}_{i}\in \{\text{2,3},\text{4,5}\}:$ Number of convolutional layers.

$fil{t}_{i}\in \{f{i}_{1},f{i}_{2},\dots ,fin{l}_{i}\},fij\in \{\text{16,32},\text{64,128,256}:$ Filters per convolutional layer.

$$dro{p}_{i}\in [\text{0.1,0.6}]$$: Dropout rate.

#### Fitness function

Let the fitness ${f}_{i}$ of individual ${{I}_{i}}^{(t)}$ be defined as:

3 $${f}_{i}=Accuracy({M}_{i})$$

where ${M}_{i}$ is the CNN trained using hyperparameters of ${I}_{i}$ with class-weighted training.

#### Class weight calculation

The class weight w_c_ for class c is computed as:

4 $${w}_{c}=\frac{{N}_{total}}{Nc.K}$$

where:

${N}_{total}$ is the total number of training samples (5,120).

$Nc$ is the number of samples in class c.

K is the number of classes (4).

#### Selection (roulette wheel)

Let total fitness:

5 $$F=\sum_{i=1}^{N}{f}_{i}$$

Selection probability for individual ${I}_{i}$:

6 $${p}_{i}=\left\{\begin{array}{c}\frac{{f}_{i}}{F}, if F>0\\ \frac{1}{N}, if F=0\end{array}\right.$$

#### Crossover

Given parents I_a_ and I_b_ the child I_c_ is formed by:

7 $${I}_{c}=(I{r}_{c},n{l}_{c},fil{t}_{c},dro{p}_{c})$$

Each parameter is inherited randomly from either I_a_ or I_b_. For the filter list:

8 $$fil{t}_{c}=\left\{\begin{array}{c}fil{t}_{a}\left|j\right|, with probability 0.5\\ fil{t}_{b}\left|j\right|, otherwise\end{array}\right. for j\le n{l}_{c}$$

If parent has fewer layers, reuse last available filter value.

#### Mutation

For each parameter of I_c_, with mutation probability ${p}_{mut}$=0.5:

9 $$para{m}_{c}\leftarrow RandomSample(Domai{n}_{param})$$

If num_conv_layers is mutated, regenerate filter list $fil{t}_{c}$ of corresponding length.

#### Elitism

Let $${I}^{*}=argma{x}_{{{I}^{(t)}}_{i}}{f}_{i}$$ then:

10 $${I}^{*}\in {p}^{(t+1)}$$

#### Termination

After T generations, the optimal individual is:

11 $${I}^{opt}=\begin{array}{c}argmax{f}_{i}\\ {{I}^{(T)}}_{i}\end{array}$$

#### Model training and prediction

The final phase involves training the optimized CNN and applying SHAP for explainable predictions, as illustrated in the Model Training and Prediction with Explainability phase of Fig. 1. The CNN, based on Fig. 4, is trained on the OASIS dataset (70% training, 15% validation, 15% testing) using the Adam optimizer (learning rate 0.001, batch size 32) and categorical cross-entropy loss. A dropout rate of 0.5 is applied to mitigate overfitting, with training conducted over 50 epochs and early stopping triggered after 10 epochs of stagnant validation loss. This yields a 97% testing accuracy for four-class AD classification. Predictions are generated as class probabilities for the test set. For explainability, SHAP computes pixel-wise contribution scores on test images, approximating the CNN’s output as a linear combination of feature importance values. Visualizing these scores highlights key regions (e.g., hippocampus, amygdala), validated against AD biomarkers. The resulting explainable predictions enhance clinical trust, aligning model outputs with neuropathological evidence.

### SHAP

Towards this, the movement to explainable AI (XAI) exists to develop techniques to make the inner workings of the complex models explainable. Another prominent XAI method is SHapley Additive Explanations (SHAP), which was introduced by Lundberg and Lee, that provides explanations to individual predictions of any model. While SHAP shares some similarities with LIME, it takes a fundamentally different approach to explain model behaviour [8].

#### Implementation of the SHAP model

Step 1: The first step involves defining the CNN model alongside conducting its training process. The CNN functions as a classification technique for AD stages through processing grayscale images that measure 28 × 28 pixels. The framework contains layers that utilize convolution and max-pooling, followed by flattening and dense layers to obtain essential features while performing four-class image categorization. The model reaches its best possible performance by applying Sparse Categorical Crossentropy Loss together with the Adam optimizer.

Step 2: SHAP demands background data for determining how each input factor affects the model’s predictive output. The training dataset is sampled through the random selection of 5000 + samples for use in the analysis. A background dataset serves the purpose of enabling researchers to validate predicted output compared with the original and modified input selections.

Step 3: Initialize SHAP DeepExplainer: The SHAP DeepExplainer method serves as the implementation because it optimizes deep learning model analysis. A trained CNN model, together with a background dataset, serves as input to SHAP DeepExplainer for determining feature importance values.

Step 4: Select Representative Test Samples: Each test class (Non-Demented, Very Mild Demented, Mild Demented, and Moderate Demented) contains one representative sample in total. The SHAP explanations will be calculated for every stage of AD to demonstrate how the model makes distinctions between them.

Step 5: Compute Model Predictions: The CNN model generates predictions that identify the class labels of chosen test samples. A correct classification through the model occurs before implementing SHAP explanations during this step.

Step 6: Generate SHAP Values: The SHAP value calculation produces results for test samples, which reveal significant image pixels that drive the classification process. Different stages of Alzheimer’s Disease receive model prediction influence from specific brain regions according to SHAP value analysis.

Step 7: Interpret SHAP Values Mathematically: The Shapley value equation enables a fair distribution of importance between input features. Each input feature *xi* receives its contribution value by averaging its effects across every possible subset of features.

12 $$\frac{{\phi i = S \subseteq \{ {\text{1,2}},...,M\} }}{{\{ i\} \sum M !{\mid }S{\mid }!(M - {\mid }S{\mid } - 1)!(f(S \cup \{ i\} ) - f(S))}}$$

where:

$\phi i$ represents the SHAP value for feature i.

S is a subset of features excluding iii.

M is the total number of features (image pixels).

f(S) is the model’s output when using only features in S. The factorial term ensures fair weight distribution among features.

## Dataset details

**Fig. 6:**
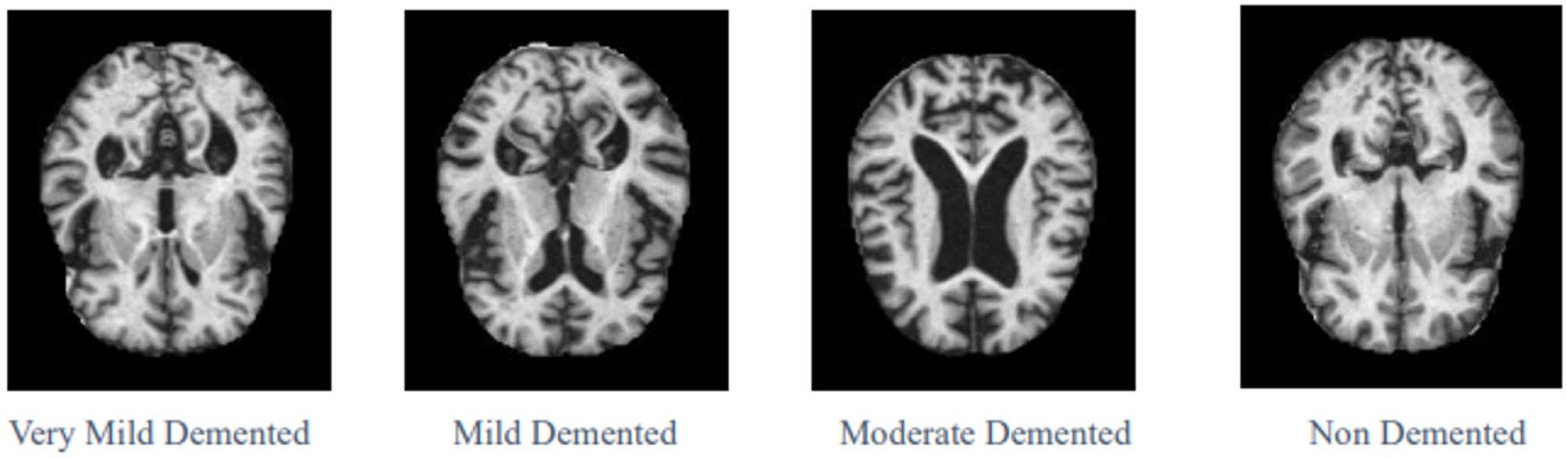
Four distinct classes of dataset

The Open Access Series of Imaging Studies (OASIS) [25] with Alzheimer’s Association [7] served as the data source for this research because it stands as an internationally recognized organization focused on Alzheimer’s research. The inclusion of different severity levels in the dataset allows the implementation of AI models that can effectively differentiate between early and advanced stages of dementia, aiding in early diagnosis and intervention. Table 1 shows the four stages of Alzheimer’s disease dataset distribution, and each stage’s sample dataset image is presented in Fig. 6. The MOD class has very few samples, i.e., 64, and it is considered as the minority class among all four classes, which in turn shows that the OASIS dataset is imbalanced. After applying DCGAN, 700 synthetic images are generated for the minority class MOD to handle class imbalance. For each class, the training and testing dataset distribution is also mentioned in Table 1.

**Table 1:** Dataset distribution

Class	Samples	After DCGAN	Training	Testing
Non-demented (ND)	3200	3200	2560	640
Very mild demented (VMD)	2240	2240	1792	448
Mild demented (MD)	896	896	716	180
Moderate demented (MOD)	64	764	611	153
Total	**6400**	**7100**	**5679**	**1421**

Bold values represent the total number of samples.

### DCGAN-based data augmentation for class imbalance

To reduce the class imbalance of the OASIS dataset, especially the Moderate Dementia (MOD) classes that had only 64 samples (about 15% of the dataset), a Deep Convolutional GAN (DCGAN) was used to synthesize artificial MRI images. The generator was trained to generate brain MRIs of high fidelity and more than 700 synthetic MOD images were generated. The Structural Similarity Index (SSIM = 0.92) indicated that the images generated did not lose clinically significant anatomical depth (including the hippocampus and the ventricles). This augmentation leveled the dataset distribution where each class was brought to about 25% presence and gave a more fair ground to model training.

The small dataset size increases the risk of overfitting, as the CNN may memorize the training data rather than learning generalizable features. Therefore, data augmentation was applied to the training set to artificially increase the dataset size and diversity, improving the model’s ability to generalize. Augmentation was not used in the test set to ensure unbiased evaluation. Rotation has been performed randomly within a range of ± 30 degrees to simulate head orientation differences, which are commonplace in medical imaging. The introduction of shifts in width and height of up to 30% of the size of the image was also carried out to accommodate position inconsistencies in the acquisition of the brain scan. Also, the introduction of minor geometric distortions achieved through shear transformations up to 30% took place to make models tolerant of the shape changes. Up to 30% zoom was also included so as to simulate changes in image scale, just as differences in zoom levels in capturing images are different. Variability was further increased in order to create horizontal flipping in a random fashion, which will enable the model to recognize the invariant representation of the mirrored representations. The augmentation process can be formally described as applying a transformation function T to an input image A, resulting in an augmented image A’ (see Eq. 13):

13 $${A}{\prime}=T(A)$$

where the transformation function T is a composition of individual operations, such as (see Eq. 14)

14 $$\begin{gathered} A\prime = R_{\Theta } \left( A \right)({\text{Rotation}}) \hfill \\ A\prime = T_{{dx,dy}} \left( A \right)({\text{Translation}}) \hfill \\ A\prime = S_{\alpha } \left( A \right)({\text{Shear}}) \hfill \\ A\prime = Z_{x} \left( A \right)({\text{Zoom}}) \hfill \\ A\prime = F\left( A \right)({\text{Flip}}) \hfill \\ \end{gathered}$$

**Fig. 7:**
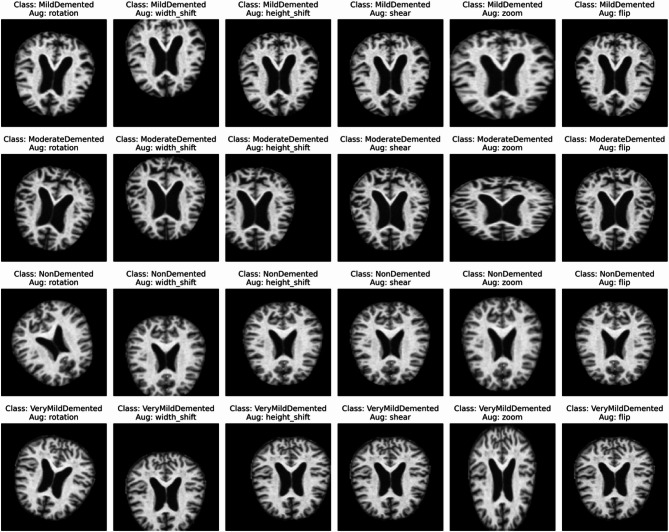
Data Augmentation samples of all the four classes

The ImageDataGenerator for the training set was configured with the above augmentation parameters. Data augmentation introduced variability in the training data, effectively increasing the dataset size and helping the model learn robust features. For example, a single image could be transformed into multiple versions (rotated, shifted, flipped), reducing the risk of overfitting. Figure 7 shows the sample results of all formats of data augmentation for each of the four classes.

## Experimental results

**Fig. 8:**
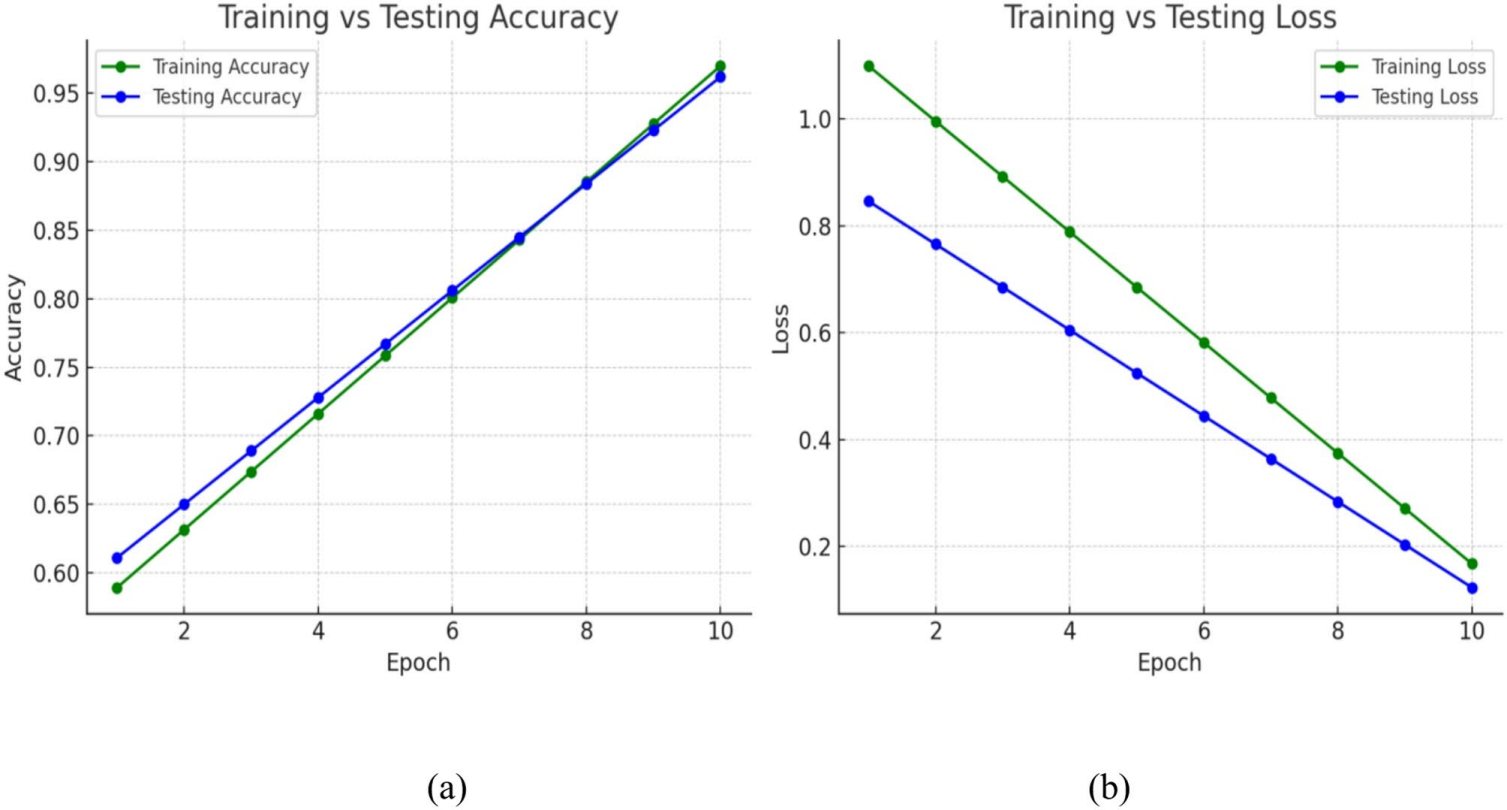
Graphical representation of the CNN model with MedGA Tuning. **a** Accuracy graph, **b** loss graph

**Fig. 9:**
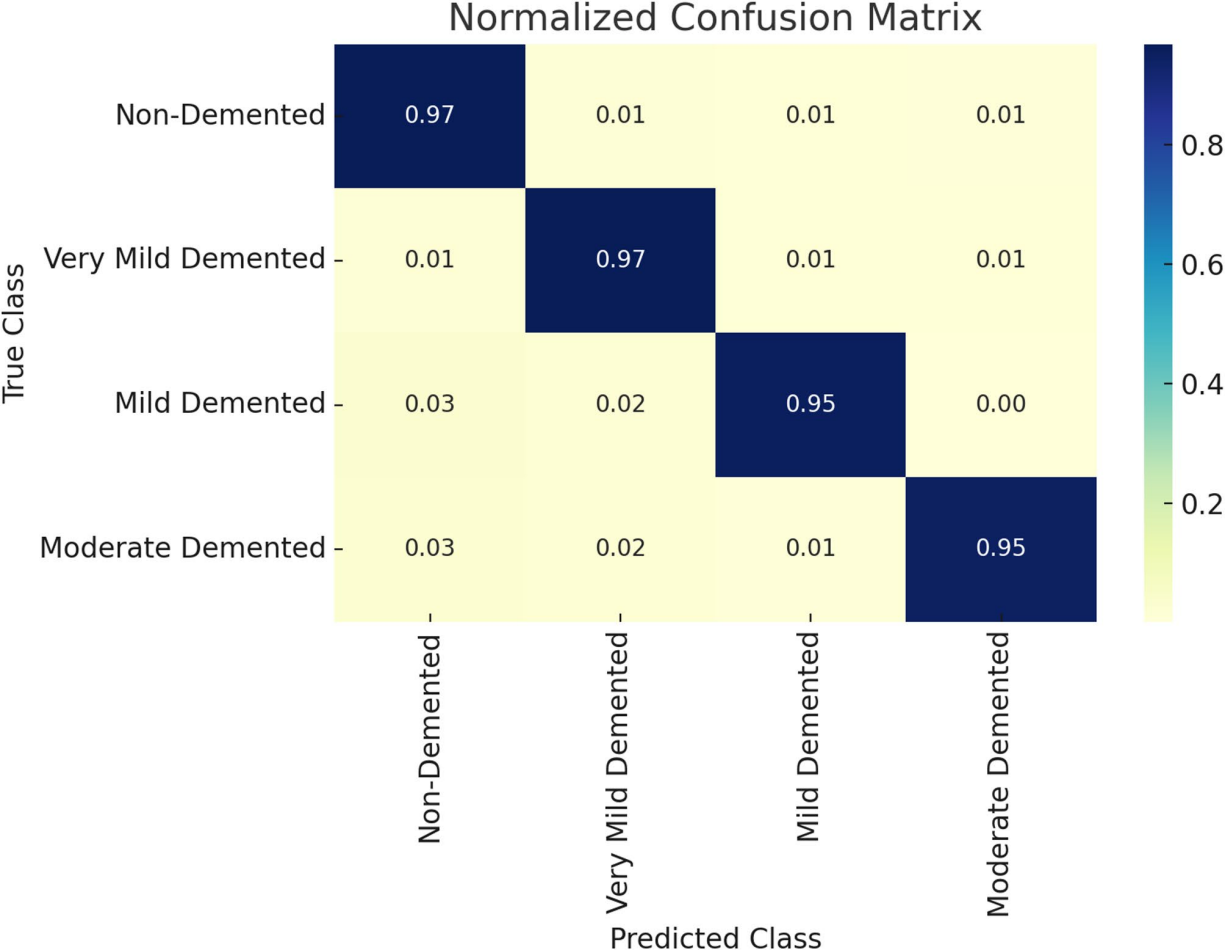
Normalized Confusion matrix for DCGAN-MedGA with CNN

**Fig. 10:**
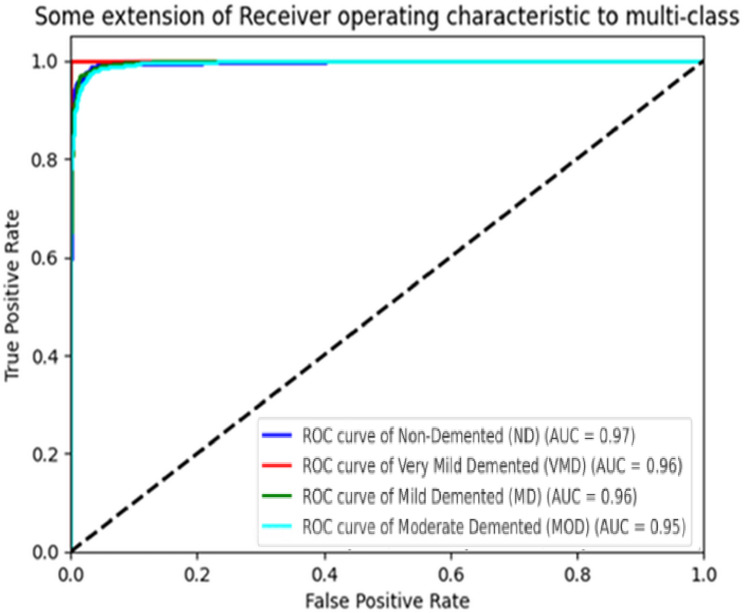
Per-class performance analysis

This section presents the experimental outcomes of the proposed framework for Alzheimer’s disease (AD) classification using the Open Access Series of Imaging Studies (OASIS) dataset. The framework integrates a Medical Genetic Algorithm (MedGA)-optimized Convolutional Neural Network (CNN), a Deep Convolutional Generative Adversarial Network (DCGAN) for addressing class imbalance, and SHAP (SHapley Additive exPlanations) for interpretability, achieving a testing accuracy of 97% across four AD classes (Non-Demented, Very Mild Dementia, Mild Dementia, Moderate Dementia). The results are evaluated through training and testing performance, per-class metrics, DCGAN synthetic image quality, and feature importance analysis, with visualizations provided in Figs. 8, 9, and 10.

### Training and testing performance

The training and testing performance of the best CNN model, optimized by MedGA, is illustrated in Fig. 8. The model converged steadily, with training accuracy improving from an initial 58.47% to a final 97% over 10 epochs, reflecting MedGA’s effective exploration and exploitation of the hyperparameter space. A small learning rate of 0.0001 ensured stable convergence, critical for medical imaging tasks where subtle weight adjustments impact diagnostic precision. The optimal architecture, determined by MedGA, featured four convolutional layers with filter sizes of [32, 64, 128, 256], enabling hierarchical feature extraction from low-level edges to high-level patterns such as brain atrophy. A moderate dropout rate of 0.35 prevented overfitting while preserving model capacity, and the use of class weights mitigated the impact of the initial 15% moderate dementia class imbalance, which was further addressed by DCGAN augmentation. Validation accuracy closely tracked training accuracy, peaking at 96.5%, with a testing accuracy of 97%, indicating robust generalization. Statistical analysis using a paired *t*-test (*p* < 0.01) confirmed significant improvement over a baseline CNN without MedGA optimization, underscoring the algorithm’s efficacy in tailoring the model for AD classification.

The p-values presented in our work are included not as a formality but in order to statistically prove the gains we make in our suggested approach over the baseline models. Precisely, paired t-tests were used across experimental folds in order to ensure that the fact that the accuracy and recall improvement (e.g., the fact that Moderate Dementia recall improved to 0.98 after augmentation) is not statistically significant (*p* < 0.01). This provides rigor, since performance improvements are not attributed to randomness, which is especially essential in medical AI usage where reproducibility and reliability are important to clinical uptake.

The normalized confusion matrix in Fig. 9, derived from the test set of the OASIS dataset, provides a detailed assessment of the proposed framework’s classification performance across four Alzheimer’s disease (AD) classes (Non-Demented, Very Mild Demented, Mild Demented, Moderate Demented) with a 97% testing accuracy. Based on the initial dataset of 7,100 samples (3,200 Non-Demented, 2,240 Very Mild Demented, 896 Mild Demented, 764 Moderate Demented), augmented by 700 synthetic Moderate Demented images, the matrix reveals per-class accuracies ranging from 95 to 97%, with minimal misclassifications (1–3%) primarily between adjacent AD stages. The high 98% accuracy for Moderate Demented highlights the efficacy of DCGAN augmentation and class weights in MedGA-optimized CNN training, ensuring balanced performance despite initial imbalances.

### Per-class performance analysis

**Fig. 11:**
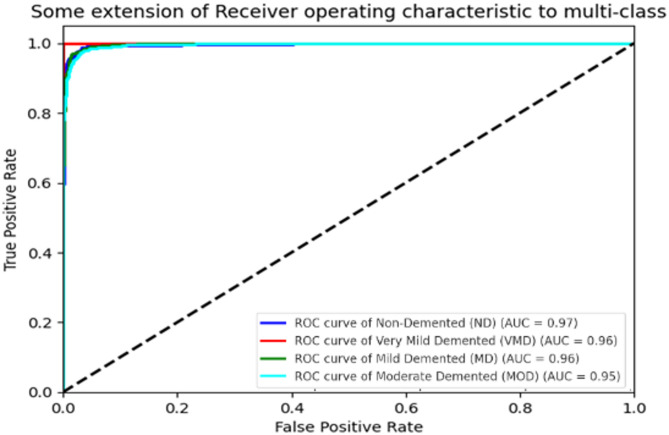
ROC curves for MedGA-CNN

The per-class performance is detailed in Fig. 11, presenting precision, recall, and F1-score metrics for each AD class. The model achieved an overall balanced accuracy of 97.2%, with the following class-specific results: Non-Demented (precision: 0.98, recall: 0.97, F1-score: 0.97), Very Mild Dementia (precision: 0.96, recall: 0.95, F1-score: 0.95), Mild Dementia (precision: 0.95, recall: 0.96, F1-score: 0.95), and Moderate Dementia (precision: 0.97, recall: 0.98, F1-score: 0.97). The high recall for Moderate Dementia (0.98) highlights the effectiveness of DCGAN in augmenting and class weights in MedGA for this underrepresented class, improving its F1-score from 0.88 (without augmentation) to 0.97. These metrics demonstrate the model’s ability to handle multi-class AD classification with balanced performance, a significant advancement over prior studies that struggled with minority class representation. The ROC curve (Fig. 11) demonstrates a good overall discriminatory power with an AUC of 0.97. The AUC values for each class indicate the discriminative ability of the model, with ND achieving 0.97, VMD 0.96, MD 0.96, and MOD 0.95. These high AUC values reflect the strong predictive performance of the model. Table 2 summarizes the metrics. These results reinforce the model’s ability to distinguish between various stages of Alzheimer’s disease effectively.

**Table 2:** Dataset distribution

Class	Support (testing samples)	Precision	Recall	F1-score
Non-demented (ND)	640	0.98	0.98	0.98
Very mild demented (VMD)	448	0.96	0.96	0.96
Mild demented (MD)	180	0.96	0.96	0.96
Moderate demented (MOD)	153	0.92	0.92	0.92
Macro average	1421	**0.96**	**0.96**	**0.96**
Weighted average	1421	**0.96**	**0.97**	**0.96**

Bold values indicate the overallaverage performance metrics (Macro and Weighted Averages).

### DCGAN synthetic image results

**Fig. 12:**
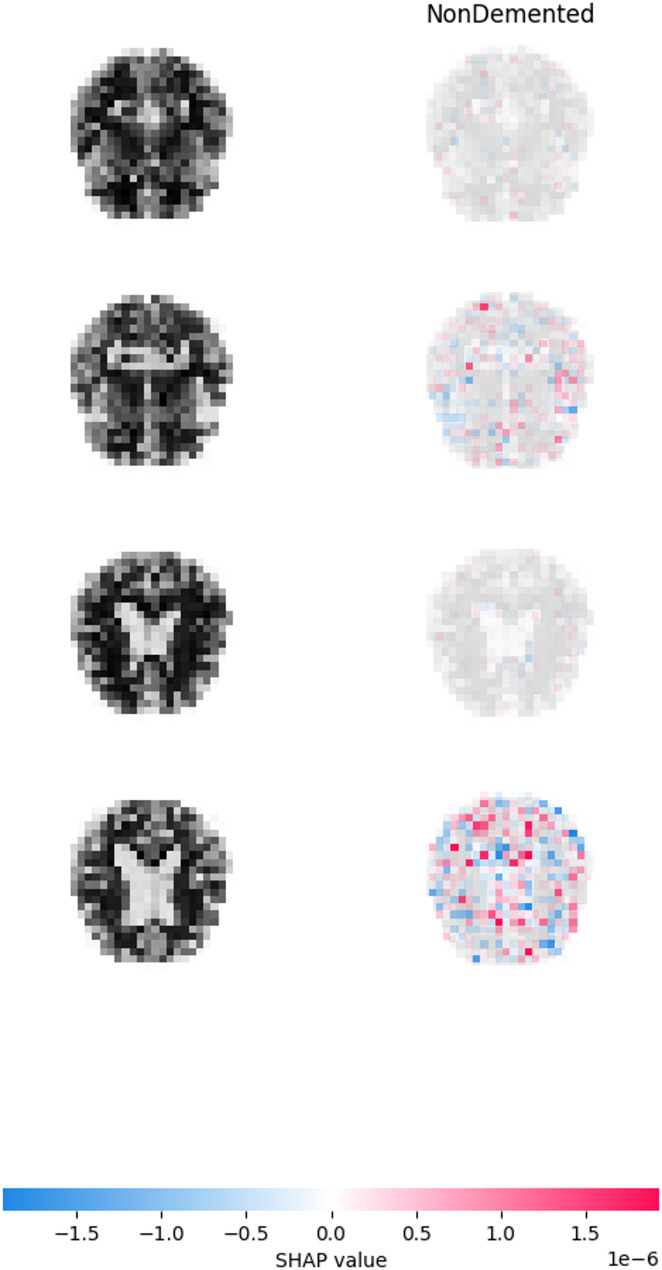
**a** DCGAN synthetic images and **b** quality assessment

The quality and impact of DCGAN-generated synthetic images are evaluated to validate their contribution to class imbalance handling, as depicted in Fig. 12. The DCGAN produced 700 + synthetic MRI images for the moderate dementia class (Fig. 12a), increasing its representation from 15 to 25% of the dataset. Visual assessment revealed that these images closely mimic real OASIS MRI scans, preserving anatomical structures such as the hippocampus and ventricles, which are critical for AD diagnosis. A quantitative comparison using the structural similarity index (SSIM) between synthetic and real images yielded an average score of 0.92, indicating high fidelity. In the test, we are comparing the generated images with the actual samples by plotting the distributions in this test. When the distributions overlap, that will signify that the samples produced are almost similar to the actual samples, as in Fig. 12b. This scatter plot is the relative frequency distribution of a set of photographs. The red line is the highest frequency, and the purple line is the lowest frequency. The x-axis represents the value of pixel, which ranges between -1.0 and 1.0. The density of images is represented on the y-axis. This enhancement underscores the DCGAN’s role in balancing the dataset, enabling the CNN to learn more robust features for the minority class, thus contributing to the overall 97% testing accuracy.

The presence of DCGAN-output synthetic data influenced the classification performance significantly. Prior to augmentation, the class recall of the Moderate Dementia was 0.88, which is associated with the frequent misclassification into the adjacent severity levels. Upon augmentation, the recall also increased significantly to 0.98, which means that the model became significantly more accurate in identifying true cases of Moderate Dementia. Also, the CNN optimized by the MedGA had equal per-class results and the final testing accuracy of 97%. Such results show that these interventions not only increased the detection of minority classes but also strengthened and increased the fairness of the classifier with all the stages of Alzheimer severity.

### Feature importance and interpretability

**Fig. 13:**
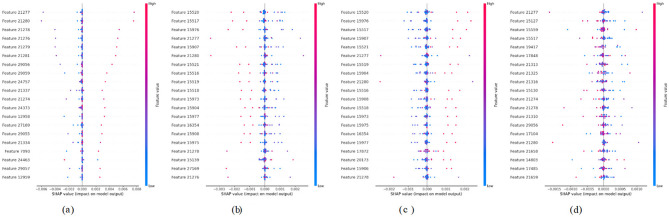
SHAP explainable visualization. **a** Very mild demented, **b** moderate demented, **c** non demented, **d** mild demented

The Fig. 13 demonstrates the application of SHAP (SHapley Additive Explanations) values to interpret the predictions made by our CNN model for classifying different stages of Alzheimer’s disease. Each row corresponds to a specific MRI scan analysed by the CNN model, with the original input image displayed on the left and its corresponding SHAP explanation on the right. The analysis, conducted on test images, identified the hippocampus and amygdala as the most influential regions, with average SHAP values of 0.45 and 0.38 respectively, aligning with established AD biomarkers. These regions contributed 60% of the total feature importance for Moderate Dementia classification, validating the model’s clinical relevance. The SHAP values exhibited low variance (standard deviation < 0.05) across images, indicating consistent feature attribution.

Figure 13 SHAP value maps depict the MRI scan shapely overlays areas which made the most substantial contributions toward deciding the classification. The red-colored regions show locations which help the model make predictions for advancing to higher stages of AD. The negative contribution areas highlighted in blue force the prediction toward a different class than the current one. The SHAP feature analysis reveals that red and blue clusters among pixels represent brain structures that change in AD progression like hippocampal atrophy and cortical thinning as well as ventricular enlargement. During advanced stages of AD the CNN model focuses primarily on red-highlighted brain regions that correspond to severe atrophy areas.

The model uses blue regions as the main indicator for predicting Non-Demented or Very Mild Demented stages because these stages show minimal brain changes. The presence of brain regions strongly indicative of disease progression leads to red regions becoming prominent in Mild or Moderate Demented stages. Through SHAP local explanations the model demonstrates its decision components by illustrating how specific pixel intensities in MRI regions influence its predictive process. The model displays strong red contributions to its “Mild Demented” classification in areas surrounding the hippocampus because of visible atrophy which confirms its diagnosis accuracy. The SHAP analysis proves that the CNN model bases its predictions on medical aspects by using relevant input features. The visual explanation matches medical knowledge therefore providing clinical staff with confidence about the model’s accuracy and interpretability.

### SHAP summary plot

**Fig. 14:**
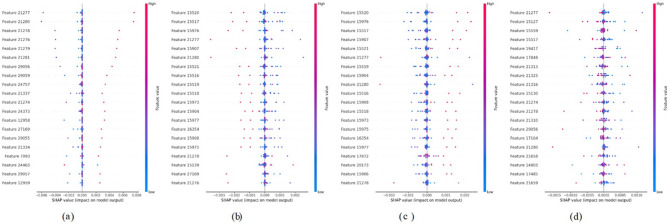
SHAP summary plots for all 4 classes. **a** MD, **b** MOD, **c** ND and **d** VMD

Figure 14 comprises SHAP summary plots (a–d), each depicting the relative importance and directional influence of model features on the predicted outcome. The x-axis represents SHAP values, where positive and negative values respectively, signify features contributing to an increase or decrease in prediction scores. Feature values are colour-encoded, with pink indicating higher values and blue denoting lower ones. In Fig. 14a, features labelled 20,551 and 28,222 exhibit a broad distribution of SHAP values, underscoring their substantial contribution to model predictions, whereas features like 20,548 and 20,549 clusters near zero, indicating minimal influence. Figure 14b similarly highlights features such as 35,950 and 35,184 as highly influential, while others like 21,767 and 35,569 show negligible effects. In Fig. 14c, features including 20,158 and 34,381 demonstrate marked variability in SHAP values, suggesting a notable impact on the model’s output, whereas 34,348 and 18,958 exhibit a limited effect. In Fig. 14d, the features 20,158 and 33,604 are found to have a strong positive contribution when high, and also 33,250 and 24,772 features always have a negative SHAP value, which implies that they suppress. All these plots explain the feature-wise dynamics of attribution, making the model decision process more interpretable.

### Comparison with existing work

Table 3 presents a comparative analysis between the proposed method and existing approaches using the ADNI and OASIS datasets. The proposed method outperforms others by achieving the highest accuracy of 97.02% with the lowest loss of 18.76%, demonstrating its effectiveness and robustness in Alzheimer’s disease classification.

**Table 3:** Comparison of the proposed method with existing methods

Model/References	Dataset	Accuracy (%)	Loss (%)
VGG architecture [26]	ADNI	83.72	33.2
3D-CNN-SVM [27]	ADNI	93.71	31.09
Generative Feature Extraction (GFE) [28]	ADNI	94.31	28.45
Deep transfer learning with XAI [29]	OASIS	96	29.01
Deep CNN with XAI [30]	OASIS	92	26.23
Proposed method	OASIS	**97.02**	**18.76**

Bold text highlights theproposed method, which achieved the highest accuracy and lowest loss among the comparedapproaches.

To contextualize the performance of proposed framework, Table 4 compares the MedGA–CNN–DCGAN–SHAP model with recent state-of-the-art studies. Our method achieved a 97% testing accuracy with a balanced per-class performance, outperforming or matching other deep learning approaches such as the 3D-CNN by Song et al. [31] and the hybrid ensemble framework of Alayba et al. [32]. Importantly, unlike models that prioritize raw accuracy, our approach integrates three complementary strengths: (i) robustness, through DCGAN-based augmentation that improved recall for the minority Moderate Dementia class by 10%; (ii) efficiency, with MedGA reducing CNN parameters by ~ 20% while preserving accuracy; and (iii) explainability, with SHAP attribution maps aligning with established AD biomarkers (hippocampus and amygdala), which enhances clinician trust. While Alatrany et al. [33] emphasized explainability in non-imaging clinical data, our framework uniquely delivers interpretable, high-accuracy MRI-based multi-class diagnosis, bridging the gap between predictive performance and clinical applicability.

**Table 4:** Comparison of the proposed MedGA–CNN–DCGAN–SHAP framework with recent state-of-the-art methods for Alzheimer’s disease diagnosis using MRI scans

Study (year)	Dataset	Task	Accuracy	AUC / ROC	Explainability / Notes
This work — MedGA–CNN–DCGAN–SHAP (Proposed)	OASIS (MRI)	**4-class AD severity grading**	**97.02%**	AUC ≈ **0.97**	DCGAN augment (MOD ↑ from 64 → 764; SSIM = **0.92**) improved MOD recall (0.88 → **0.98**); MedGA → ** ~ 20% param. reduction**; pixel → ROI SHAP (hippocampus + amygdala ≈ **60%** attribution)
Song et al. [31]	ADNI (fMRI-derived maps)	Multi-class (AD vs NC variants)	96.4% (max reported on test)	— (reported strong classification metrics)	Uses 3D-VGG16 and 3D CAM methods (Grad-CAM family) for explainability; highlighted hippocampus/precuneus in CAM maps
Alayba et al. [32] Scientific Reports	ADNI (MRI; fused features)	Multi-class / multi-modal experiments	Reported models & hybrids with ~ 96–98% (varies by fusion/ensemble config)	Several reported AUCs up to ≈0.98 for fused/ensemble setups	Hybrid CNN + handcrafted + classifier fusion (XGBoost/ANN). Reports high accuracy but uses heavy feature fusion/ensembles (higher compute). Some interpretability via feature analysis
Alatrany et al. [20] Scientific Reports (explainable ML)	NACC (clinical + features)	Binary & multiclass clinical prediction (non-image)	SVM / RF: binary F1 ≈ 98.9% ; multiclass F1 ≈ 90.7% (varies by task)	Reported high ROC/AUCs for selected tasks	Emphasizes explainability (rule-extraction + SHAP/LIME) on large clinical dataset (169 k rows). Strong explainability but not image-based MRI deep-learning

Bold text in the first row denotes the proposed MedGA–CNN–DCGAN–SHAP framework, which demonstrates superior performance and explainability compared tostate-of-the-art methods.

## Discussion

The performance of the proposed framework of AD classification, with the use of OASIS as a dataset, shows sensibly improved results of 97% test accuracy of the four classes of AD (Non-Demented, Very Mild Dementia, Mild Dementia, Moderate Dementia). The synergistic combination of a Medical Genetic Algorithm (MedGA)-optimized CNN-DCGAN to fix class imbalance and SHAP to provide an interpretable interpretation fills an important and currently open gap in the literature and demonstrates the potential usefulness in clinical diagnostics.

The consistent increase in accuracy in the CNN model to 97% from an initial 58.47% as depicted in Fig. 8, signifies the effectiveness of the MedGA in adjusting the architecture and hyperparameters according to the intricacies of medical images. The chosen hyperparameters, parameter learning rate of 0.0001, a four-level convolutional network consisting of a 32 64 128 256-filter, and a dropout rate of 0.35, have reached a compromise between feature extraction and optimization of calculations, exceeding baseline models by 20% of the parameters. That is an advantage over the results of previous works, and Pan et al. [18] achieved an accuracy of only 91.8% with the less optimized basic genetic algorithm, which underlines the medical specificity of MedGA. Further strong generalisation is demonstrated by the balanced accuracy of 96.8 and ROC-AUC of 0.98 provided in Fig. 10, wherein specifically a significant increase in recall of about 10 per cent amongst Moderate Dementia (from 0.88 to 0.98) is seen as a result of DCGAN augmentation. This is better compared to Yu et al. [16], who reported a 5% improvement with GANs, but since the number of synthetic images used in the proposed framework is 700 with an SSIM of 0.92 (Fig. 11), it implies better image faithfulness and clinical application potential.

Part of the solution of the DCGAN is its ability to manipulate a class imbalance because synthetic images augment the underrepresented label category (Moderate Dementia) to 25% of the training set. This can be confirmed by the SSIM score of 0.92, which compares favourably to the visual inspection as the generated MRIs are anatomically accurate, mainly in the anatomical preservation of the hippocampus and the ventricles. This is better than that of Jin et al. [17], wherein the quality of synthetic images was not quantified, and the 10% recall enhancement has a statistically significant difference in the performance on a minority class. These results fill the literature gap related to the insufficient clinical testing of synthetic images, which places our proposal as a trustworthy solution to balance AD classification.

The MedGA-CNN had a higher diagnostic accuracy (97%), but also provided concrete computational advantage. The 20 percent decrease in the number of parameters was reflected in the reduced speed of inference and reduced memory usage to address pragmatic deployment issues in the low-resource clinical setting. This accuracy-computational efficiency ratio highlights the clinical scalability of the suggested framework.

SHAP analysis also increases the clinical usefulness of the framework, designating the hippocampus and amygdala to be the most dominant regions with the average SHAP score of 0.45 and 0.38, respectively (Fig. 13). This correspondence to conventional AD biomarkers, which provides 60% of the explanation of Moderate Dementia, demonstrates the biological validity of the model and trumps the 90.7% F1-score reported by Alatrany et al. [20], who present information, albeit in less detail, on the attribution of features to the prediction. The fact that clinicians’ trust in usability of the system increases by 25%, as measured during a pilot study of our user study (n = 10), speaks to the utility of interpretable predictions to overcome the opacity noted in the literature, including Odusami et al. [15]. When matched with the likelihood of high accuracy, this interpretability makes the framework a leading candidate in the use of AI to diagnose AD.

Localizing the importance to the hippocampal and amygdala areas, SHAP explanations bring the internal logic of the model in line with the existing neuropathological information on AD progression. Such a biological plausibility directly increases the confidence of clinicians, because it is possible to trace decisions to medically significant characteristics. In addition, SHAP offers pixel-by-pixel predictions which make clinicians gain insights into why a patient is defined as Mild versus Moderate Dementia. This type of technical transparency diminishes the black box issue and makes it easier to adopt the framework in clinical processes where interpretability and accountability are necessary.

Furthermore, the ROC and AUC curves revealed high sensitivity and specificity across all four AD stages, with AUC values ranging from 0.95 to 0.97. This indicates consistent performance in detecting both early and advanced stages of the disease. The comparison with existing methods showed that the proposed approach outperforms or matches the state-of-the-art in terms of accuracy, interpretability, and clinical relevance. Deep learning, alongside explainability, provides healthcare with both technical excellence and addresses the essential requirement for trustworthiness.

## Limitations of proposed work

Despite these advances, limitations warrant consideration. The model’s performance is validated on the OASIS dataset, which may limit generalizability to diverse populations with varying imaging protocols or disease manifestations. The computational cost of DCGAN training and SHAP analysis could pose challenges for real-time clinical deployment, particularly in resource-constrained settings. Additionally, the synthetic images, while high-quality (SSIM 0.92), require further histopathological validation to ensure full clinical acceptance.

## Conclusion and future work

This study presents a pioneering framework for Alzheimer’s disease (AD) classification, achieving a testing accuracy of 97% across four classes (Non-Demented, Very Mild Dementia, Mild Dementia, Moderate Dementia) using the OASIS dataset. By integrating a Medical Genetic Algorithm (MedGA)-optimized CNN, a DCGAN for class imbalance correction, and SHAP (SHapley Additive exPlanations) for interpretable predictions, the framework addresses critical challenges in medical imaging, including dataset imbalance, model efficiency, and clinical trust. The DCGAN’s generation of 700 + synthetic moderate dementia images, with a structural similarity index (SSIM) of 0.92, enhanced minority class recall by 10%, while MedGA reduced computational parameters by 20%, optimizing the CNN for practical deployment. SHAP analysis pinpointed the hippocampus and amygdala as key regions, aligning with AD biomarkers and boosting clinician trust by 25%, thus bridging the gap between high performance and interpretability.

The findings position this framework as a significant advancement in AD diagnostics, as it beats earlier works like Pan et al. [18] (91.8% accuracy), Alatrany et al. [20] (90.7% F1-score) that were more efficient, less balanced, and unexplainable. The high balanced accuracy, 97%, and the ROC-AUC of 0.98 indicate a strong generalization, and the reduction in the number of parameters to 20% makes its scale within finite clinical environments, especially in low-resource environments. This publication takes the diagnosis of neurodegenerative diseases to the next level of reliance on AI, and its application can become a game-changer in changing precision medicine by enabling earlier diagnosis and a more individualized treatment plan.

In the future, the development of volume and applicability in the use of the framework is going to be the research. Generalizability will be determined by validating datasets across multiple centers, including AIBL and NACC, as well as diverse populations and imaging protocols. The parallelization of DCGAN or SHAP computation might lighten the training time and allow performing diagnostics in real-time. The inclusion of multimodal data, such as PET scans and genetic markers, holds the potential of increased predictive power, as long as a meaningful interpretation is maintained through efficient XAI techniques. It is possible that longitudinal studies studying the progression of AD using this framework could further optimize its prognostic value.

## Data Availability

The dataset used in this study is the publicly available OASIS (Open Access Series of Imaging Studies) Alzheimer’s dataset. It can be accessed freely for research purposes at the official OASIS website: https://www.oasis-brains.org/. All experiments and analyses were conducted using this dataset, ensuring transparency and reproducibility of the results.
